# Noninferiority of Ultrasound‐Guided Modified Intercostal Block to Traditional Approach for Analgesia After Minimally Invasive Repair of Pectus Excavatum in Children: A Randomized Trial

**DOI:** 10.1155/prm/2183328

**Published:** 2026-03-14

**Authors:** Ming-Wen Yang, Xian-Lun Duan, Yu-Zhu Cai, Yin Xia, Ying-Ying Sun, Xin-Qi Cheng

**Affiliations:** ^1^ Department of Anesthesiology, First Affiliated Hospital of Anhui Medical University, 218 Jixi Road, Hefei, 230000, Anhui, China, ahtcm.edu.cn; ^2^ Department of Anesthesiology, Anhui Provincial Children’s Hospital, 39 Wangjiang East Road, Hefei, 230000, Anhui, China, ahetyy.com; ^3^ Department of Pediatric Thoracic Surgery, Anhui Provincial Children’s Hospital, 39 Wangjiang East Road, Hefei, 230000, Anhui, China, ahetyy.com

**Keywords:** funnel chest, minimally invasive surgical procedures, nerve block, pain, pediatric, postoperative, ultrasonography

## Abstract

**Background:**

For pediatric pectus excavatum, the standard treatment is minimally invasive repair of pectus excavatum (MIRPE). A major challenge, however, is the severe postoperative pain. Although ultrasound‐guided intercostal nerve block (UINB) offers effective analgesia, the technique’s complexity and associated safety concerns are significant barriers, deterring its routine use. Modified intercostal nerve block (MINB) is effective in adult thoracic surgery but unvalidated in pediatric MIRPE.

**Objective:**

To evaluate MINB’s noninferiority to UINB for postoperative analgesia and safety in children undergoing MIRPE.

**Design:**

Single‐center randomized noninferiority trial.

**Methods:**

Seventy‐six ASA I–II pediatric patients (8–18 years) scheduled for single‐bar MIRPE were 1:1 randomized to the MINB or UINB group. Primary outcome includes 24‐h postoperative coughing visual analog scale (VAS) score (noninferiority margin Δ = 1.0). Secondary outcomes include resting/coughing VAS scores at 3, 6, 9, 12, 24, and 48 h postoperatively; procedure duration; local anesthetic dose; needle complications; opioid consumption; rescue analgesia; and adverse events.

**Results:**

The mean difference in 24‐h coughing VAS (MINB‐UINB) score was −0.02 (95% CI: −0.85 to 0.80), confirming noninferiority of MINB (upper 95% CI limit 0.80 < noninferiority margin Δ = 1.0). MINB reduced procedure time by 65% (4.6 ± 1.3 vs. 13.2 ± 1.6 min; *p* < 0.001), decreased ropivacaine dose by 19% (50.0 ± 0.0 vs. 61.9 ± 4.6 mg; *p* < 0.001), shortened anesthesia duration (119.6 ± 18.3 vs. 131.8 ± 14.6 min; *p* = 0.002), and eliminated vascular injuries (0% vs. 16.2%; *p* = 0.025). All other outcomes demonstrated no statistically significant differences in the comparisons between the groups (*p* > 0.05).

**Conclusions:**

For children undergoing single‐bar MIRPE, MINB provides noninferior analgesia to UINB with critical advantages: 65% faster placement, 19% lower ropivacaine dose, reduced anesthesia duration, and elimination of vascular injuries. These findings suggest that MINB offers a valuable alternative to UINB for post‐MIRPE analgesia, as it appears to provide a more favorable balance between safety and efficiency.

**Trail Registration:** Chinese Clinical Trial Registry: ChiCTR2200057961

## 1. Introduction

Pectus excavatum (PE) is the most prevalent congenital chest wall deformity, with an estimated incidence of 1 in 300–400 live births [1]. The minimally invasive repair of pectus excavatum (MIRPE) is the established standard for surgically correcting PE, and this technique is also widely known as the Nuss procedure [1–4]. Despite its minimally invasive nature, evidence indicates that pediatric patients undergoing MIRPE may experience more severe postoperative pain than those receiving open repair, necessitating effective analgesia [5, 6].

Effective postoperative analgesia following MIRPE can be achieved through modalities such as thoracic epidural analgesia (TEA), patient‐controlled intravenous analgesia (PCIA), and intercostal nerve cryoablation (CA) [1–3, 7]. However, each technique has limitations: A retrospective study reported catheter insertion failure or dysfunction in 35% of pediatric patients receiving TEA following MIRPE, and TEA carries a risk of needle‐related nerve injury [3, 7–9]; PCIA requires high‐dose opioid administration, which can trigger adverse effects such as nausea, vomiting, pruritus, and even respiratory depression [1, 10, 11]; CA is associated with prolonged surgical duration, elevated costs, and potential cutaneous sensory deficits on the chest wall [4, 7, 9, 12].

The advancement of ultrasound has propelled ultrasound‐guided regional blocks to the forefront of pain management. By integrating these blocks into multimodal analgesia that combines PCIA with nonopioid agents (e.g., tramadol, NSAIDs), clinicians can achieve potent analgesia while simultaneously reducing opioid dependence and its adverse effects [13, 14]. Ultrasound‐guided intercostal nerve block (UINB) precisely targets the intercostal nerves, which are the primary pathways for postoperative pain after thoracic surgery [13, 15]. A meta‐analysis indicates that intercostal nerve block offers analgesic efficacy comparable to TEA within the first 24‐h post‐thoracic surgery [16]. UINB can be conducted entirely with the patient supine, thereby eliminating the requirement for repositioning into prone or lateral decubitus [17]. Supported by evidence, UINB provides dual benefits of effective analgesia and opioid reduction in MIRPE [9, 13], and it remains the primary regional technique at our institution. However, its clinical application faces several practical challenges: The procedure requires multiple punctures, carries pleural injury risk due to the needle trajectory, and often depends on high volumes of local anesthetic [18].

We report a modified intercostal nerve block (MINB), developed to optimize conventional UINB, which has been validated in our prior adult studies [19–21]. Briefly, the MINB technique entails a single injection into a specific anatomical potential space near the thoracic incision. This space is defined by the inferior margin of the rib, with the intercostal muscles superficial to it and the serratus anterior muscle deep to it, allowing for a combined intercostal nerve and serratus plane block using a low volume of local anesthetic [21]. Although MINB has demonstrated opioid‐sparing effects and reduced postoperative pain scores in adults undergoing minimally invasive thoracic surgery (MITS) [19, 20], its efficacy for analgesia in pediatric patients undergoing MIRPE remains unknown. To address this gap, we designed and conducted a randomized noninferiority trial to test our hypothesis: that for pediatric patients undergoing MIRPE, MINB would offer analgesic efficacy noninferior to UINB, while also yielding benefits in procedural ease, decreased anesthetic requirements, and fewer complications.

## 2. Methods

### 2.1. Trial Design and Patients

This randomized, noninferiority trial was approved by the Medical Ethics Committee of Anhui Provincial Children’s Hospital (Approval No: EYLL‐2022–039) (Date of Registration: March 24, 2022). Before enrollment into the study, written informed consent was acquired from the parents or legal guardians of every participant. Eligible patients were pediatric individuals aged 8–18 years with American Society of Anesthesiologists (ASA) physical status I or II, diagnosed with PE, and scheduled for elective single‐bar MIRPE under general anesthesia. All surgical procedures in this study were conducted by the same surgical team at our institution over the period from September 1, 2022, to December 31, 2023. Exclusion criteria encompassed: known allergy to local anesthetics; coagulopathy; active infection at potential puncture sites; chronic opioid or acetaminophen use; cognitive impairment precluding visual analog scale (VAS) assessment; guardians unable to manage PCIA; or refusal to participate. Patients experiencing intraoperative surgical plan change or incomplete data collection were also excluded from the final per‐protocol analysis.

### 2.2. Randomization and Blinding

All eligible pediatric patients were randomly allocated in a 1:1 ratio to the MINB or UINB group by an independent investigator, employing a computer‐generated simple randomization sequence from SPSS (v22.0, USA) with equal allocation and no stratification or matching. An independent investigator secured the group assignments in sequentially numbered, sealed, opaque envelopes and opened each one only immediately prior to performing the nerve block. All block procedures were performed in the induction room after endotracheal intubation. Uninvolved operating room staff exited the induction room before block preparation. Local anesthetic solutions were prepared and administered by a dedicated anesthesiologist not involved in subsequent care. After block completion, puncture sites were covered with sterile dressings to maintain allocation concealment. Subsequently, patients were taken to the operating room for the surgical procedure. The physicians performing randomization and nerve blocks took no part in intraoperative management, postoperative follow‐up, or data analysis. All other clinical staff, outcome assessors, and statisticians remained blinded to group assignments throughout the trial.

### 2.3. Management of Anesthesia

The standardized intraoperative monitoring protocol comprised electrocardiogram (ECG), pulse oximetry (SpO_2_), noninvasive blood pressure (NIBP), and bispectral index (BIS) monitoring. Patients received sufentanil (0.4 μg/kg), propofol (3.0 mg/kg), and cisatracurium (0.15 mg/kg) for anesthetic induction, followed by endotracheal intubation. Anesthesia was maintained using sevoflurane at 1–1.5 minimum alveolar concentration (MAC), which was adjusted to maintain BIS values between 40 and 60. Hemodynamic parameters were maintained within 20% of preoperative baselines through adjustments in anesthetic depth, phenylephrine boluses (1–2 μg/kg), or Ringer’s lactate solution boluses (10–20 mL/kg) as required. Supplemental intraoperative analgesia was provided using remifentanil infusions (initial 0.1 μg/kg/min, titrated ≤ 0.3 μg/kg/min). At the conclusion of surgery, patients received an intravenous injection of ondansetron (0.1 mg/kg) and sufentanil (0.1 μg/kg), after which the PCIA pump was connected. Tracheal extubation occurred after confirming adequate spontaneous ventilation and eye opening, with transfer to the postanesthesia care unit (PACU).

All pediatric patients received postoperative analgesia via a standardized PCIA regimen, which consisted of a solution of sufentanil (3.0 μg/kg) and tropisetron (0.1 mg/kg) in 150 mL of normal saline, administered via a pump set for a 2‐mL/h background infusion, 2‐mL bolus doses, and a 15‐min lockout period. Guardians were trained in standardized PCIA operation by dedicated nursing staff. Rescue analgesia with intravenous tramadol (2 mg/kg) [22] was administered when pain persisted (VAS score ≥ 4 after three consecutive boluses) or for severe breakthrough pain (VAS score ≥ 7).

### 2.4. Nerve Block

Following anesthetic induction, patients were positioned supine. Subsequently, the puncture site was prepared using standard antiseptic techniques.

**FIGURE 1:**
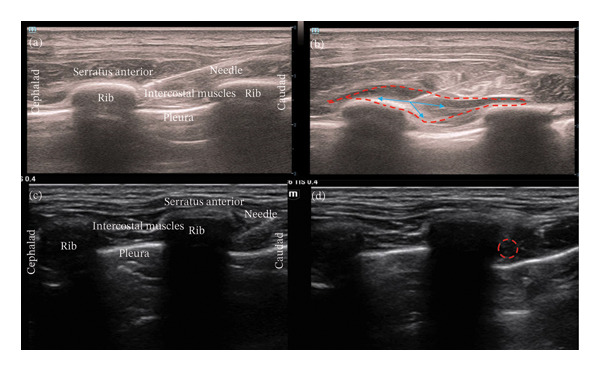
Ultrasound images of MINB preinjection (Figure (a) and postinjection (Figure (b)); ultrasound images of UINB preinjection (Figure (c)) and postinjection (Figure (d)). Notes: Blue arrows indicate the direction of local anesthetic spread; red dashed line delineates the spread area. Abbreviations: MINB, ultrasound‐guided modified intercostal nerve block; UINB, ultrasound‐guided intercostal nerve block.

**MINB:** A high‐frequency linear ultrasound probe (L14‐5wu; Resona 7EXP; Mindray, China) was employed to perform the MINB procedure. The probe was positioned longitudinally along the intercostal space, 2–3 cm lateral to the planned surgical incision. Under real‐time ultrasound imaging, the serratus anterior muscle, ribs, intercostal muscles, and pleura were identified. Using an in‐plane ultrasound‐guided technique, a 20‐gauge needle was advanced in a caudad‐to‐cephalad direction with the bevel oriented downward to facilitate intercostal spread of local anesthetic until its tip reached the fascial plane at the intersection of the deep surface of the serratus anterior muscle and the inferior margin of the rib. Following hydrodissection with a small volume of normal saline to confirm needle‐tip position, 10 mL of 0.25% ropivacaine was administered. Real‐time ultrasound was used to verify the appropriate spread of local anesthetic, showing craniocaudal distribution deep to the serratus anterior muscle and downward diffusion toward the intercostal muscles (downward displacement of the pleura) (Figures 1(a), 1(b)), indicating successful MINB implementation. An identical block was then administered on the contralateral side.

**UINB:** The UINB procedure was carried out based on the technique established by Luo et al. [10]. Briefly, under ultrasound guidance at the midaxillary line, a 20‐gauge needle was inserted in a caudal‐to‐cephalad direction targeting the plane superficial to the pleura. After confirmation of proper needle placement, 0.25% ropivacaine was injected (dose stratified by weight: ≤ 15 kg: 0.2 mL/kg; 15–30 kg: 3 mL; 30–45 kg: 4 mL; ≥ 45 kg: 5 mL), to achieve longitudinal spread along the intercostal muscles and downward displacement of the pleura (Figures 1(c), 1(d)). A total of six needle insertions were performed bilaterally at three intercostal levels: the intercostal space at the incision level, the intercostal space immediately superior to the incision, and the intercostal space immediately inferior to the incision.

### 2.5. Outcomes

Since pain after MIRPE is frequently aggravated during coughing or deep breathing, adequate coughing is critical for secretion clearance and prevention of pulmonary complications in children. The predefined primary outcome was coughing‐induced pain intensity assessed at 24 h postoperatively using a 10‐cm VAS (0 = no pain, 10 = worst imaginable pain) [23]. Secondary outcomes included the following: (1) resting and coughing VAS scores at 3, 6, 9, 12, 24, and 48 h postoperatively; (2) block procedural metrics (success rate, performance duration, total ropivacaine dose); (3) the total postoperative sufentanil consumption via PCIA over 48 h, along with the number of PCA demands, the time to the first analgesic request, and the requirement for tramadol rescue; (4) adverse events, including vascular puncture (defined as the thoracoscopic observation of a pleural hematoma without discontinuity of the pleural lining), pleural injury (defined as the thoracoscopic observation of discontinuity of the pleural lining), and postoperative nausea and vomiting (PONV); (5) length of stay in PACU and hospital. We assessed patient satisfaction regarding their analgesia at the time of discharge, using a 10‐point scale. PONV severity was documented within 48 h using a 4‐point scale (0 = none, 1 = mild nausea, 2 = severe nausea/single emesis, 3 = recurrent vomiting) [24]. All VAS assessments were conducted by nurses blinded to group allocation and trained in outcome assessment.

### 2.6. Statistical Analysis

Sample size determination focused on the primary endpoint (coughing VAS score at 24 h postoperatively). Data from a preliminary study of 20 patients (10 per group) demonstrated mean ± standard deviation (SD) VAS scores of 4.03 ± 0.65 for the MINB group and 3.75 ± 1.11 for the UINB group. The noninferiority margin (Δ) was prespecified as 1.0 cm, based on a prospective observational study published in the *British Journal of Anaesthesi*a (2017) [25]. This study systematically determined the minimal clinically important difference (MCID) for postoperative pain VAS scores by integrating anchor‐based and distribution‐based methods (including 0.3 SD, standard error of measurement, and 5% range) and confirmed that a 9.9‐mm (rounded to 10 mm, i.e., 1.0 cm) change in the 100‐mm VAS score signifies a clinically meaningful improvement or deterioration in pain status. Notably, the study explicitly emphasized that the MCID of the VAS score can be used to guide the setting of noninferiority margins in clinical trials, providing direct methodological support for our prespecified margin [25]. Based on the primary outcome and using PASS (v15.0; USA) with settings of 80% power and a one‐sided significance level of 0.025 (*α* = 0.05 for a two‐sided noninferiority test), the initial sample size was calculated to be 54 patients. This was increased to 68 to allow for a projected 20% attrition rate.

Data analysis utilized SPSS (v22.0, USA) and GraphPad Prism (v9.0, USA). Normally distributed continuous variables (confirmed by Shapiro–Wilk test/histogram inspection) were expressed as mean ± SD with between‐group comparisons via independent *t*‐tests (95% CI for mean differences). Non‐normal continuous data were reported as median (IQR) and analyzed using Mann–Whitney *U* tests (95% CI for median differences via Hodges–Lehmann). Categorical variables presented as frequencies (%) underwent *χ* ^2^ or Fisher’s exact testing. Statistical significance was defined as *p* < 0.05.

Noninferiority of MINB versus UINB regarding the primary endpoint was declared when the upper limit of the 95% CI for the difference (MINB‐UINB) in 24‐h cough VAS scores did not exceed the predefined margin of 1.0 cm.

## 3. Results

**FIGURE 2:**
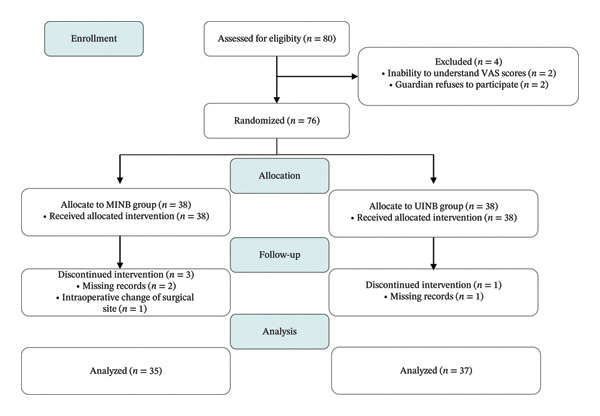
Consort flow diagram. Abbreviations: MINB, ultrasound‐guided modified intercostal nerve block; UINB, ultrasound‐guided intercostal nerve block; VAS, visual analog scale.

Eighty patients were screened for eligibility. Of these, 76 met the inclusion criteria and were randomized to the MINB group (*n* = 38) or the UINB group (*n* = 38). Four patients were excluded after randomization due to incomplete outcome data (*n* = 3) or intraoperative modification of the surgical plan (*n* = 1). The final per‐protocol analysis cohort comprised 72 patients (MINB group: *n* = 35; UINB group: *n* = 37) (Figure 2).

### 3.1. Baseline Characteristics

Table 1 displays the demographic and intraoperative characteristics of the study participants. Anesthesia duration was significantly shorter in the MINB group than in the UINB group (119.6 ± 18.3 min vs. 131.8 ± 14.6 min; mean difference, −12.3 min; 95% CI: −20.0 to −4.5; *p* = 0.002). No significant between‐group differences were observed for the remaining variables (*p* > 0.05).

**TABLE 1:** Patients’ demographic characteristics and intraoperative variables.

Variables	MINB group (*n* = 35)	UINB group (*n* = 37)
Age (years)	13 (12–14)	13 (12–14)
Sex (male/female)	33/2	34/3
BMI (kg/m^2^)	22.3 ± 2.3	21.9 ± 2.8
ASA class (I/II)	17/18	23/14
Haller index	3.2 ± 0.3	3.0 ± 0.3
Surgical incision level (ICS5‐8)	6/12/10/7	8/11/13/5
Duration of surgery (mins)	83.9 ± 9.4	86.2 ± 11.3
Duration of anesthesia (mins)	119.6 ± 18.3	131.8 ± 14.6^∗^
Extubation time	15.8 ± 4.0	17.2 ± 3.5
Length of PACU stay (mins)	37.1 ± 4.2	35.6 ± 3.5
Intraoperative sufentanil dose (μg)	20.4 ± 3.5	21.7 ± 3.2
Intraoperative remifentanil dose (μg)	634.1 ± 145.3	598.2 ± 134.8
Hospital length of stay (days)	6 (5–7)	6 (5–7)

*Note:* Data are expressed as mean ± SD, median (IQR), or number of patients.

Abbreviations: ASA, American Society of Anesthesiologists; BMI, body mass index; ICS, intercostal space; MINB, ultrasound‐guided modified intercostal nerve block; PACU, postanesthesia care unit; UINB, ultrasound‐guided intercostal nerve block.

^∗^Statistical significance (*p* < 0.05).

### 3.2. Primary Outcome

**FIGURE 3:**
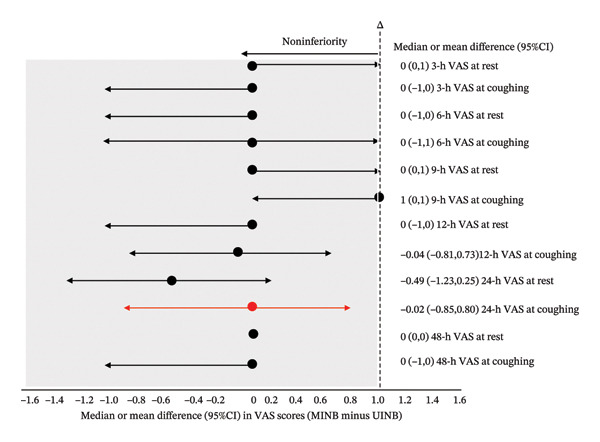
Noninferiority analysis of VAS scores between groups across time points. Notes: Data are plotted as differences in the means or median of the MINB group minus the UINB group with 95%CI; vertical dashed lines demarcate the prespecified noninferiority margin (Δ = 1); gray‐shaded areas indicating the noninferiority zone; red circles and lines represent the median and 95% CI of the between‐group difference in VAS scores at 24 h postoperatively for the primary outcome. Abbreviations: CI, confidence interval; Δ, margin of noninferiority; VAS, visual analog scale; MINB, ultrasound‐guided modified intercostal nerve block; UINB, ultrasound‐guided intercostal nerve block.

The 24‐h postoperative cough VAS score was 3.57 ± 1.82 in the MINB group versus 3.59 ± 1.69 in the UINB group (mean difference, −0.02; 95% CI: −0.85 to 0.80). Noninferiority was confirmed because the upper limit of the 95% confidence interval (0.80) was below the prespecified noninferiority margin (Δ = 1.0) (Figure 3). The noninferior analgesic efficacy of MINB persisted across all time points (Figure 3).

### 3.3. Secondary Outcomes

Postoperative resting pain (Figure 4(a)) and coughing pain (Figure 4(b)) VAS scores over 48 h are presented in Figure 4. Analyses showed no significant differences between the groups at all time points (*p* > 0.05). Both groups demonstrated significant increases in pain scores over time, with VAS scores for resting and coughing pain significantly elevated at 9, 12, 24, and 48 h compared to the 3 h (*p* < 0.01, Bonferroni correction). Secondary outcomes are summarized in Table 2. Puncture time was reduced by 65% in the MINB group compared with the UINB group (4.6 ± 1.3 min vs. 13.2 ± 1.6 min; mean difference, −8.6 min; 95% CI: −9.22 to −7.84; *p* < 0.001). Ropivacaine consumption was 19% lower in the MINB group (50.0 ± 0.0 mg vs. 61.9 ± 4.6 mg; mean difference, −11.9 mg; 95% CI: −14.42 to −10.34; *p* < 0.001). The incidence of vascular injury was significantly lower with MINB than with UINB (0% vs. 16.2%; *p* = 0.025), while pleural injury demonstrated a nonsignificant trend toward reduction (0% vs. 8.1%; *p* = 0.240). No significant between‐group differences were observed for the remaining secondary outcomes (*p* > 0.05).

FIGURE 4The VAS scores at rest (a) and at coughing (b) at each time point after surgery in the two groups. Notes: Boxes represent IQR with median line; whiskers indicate minimum and maximum values. ^∗^Statistically significant difference versus the 3‐h time point within the MINB group (*p* < 0.01); ^#^Statistically significant difference versus the 3‐h time point within the UINB group (*p* < 0.01) (Wilcoxon test with Bonferroni correction). Abbreviations: VAS, visual analog scale; MINB, ultrasound‐guided modified intercostal nerve block; UINB, ultrasound‐guided intercostal nerve block; ns, not significant.

**Figure:**
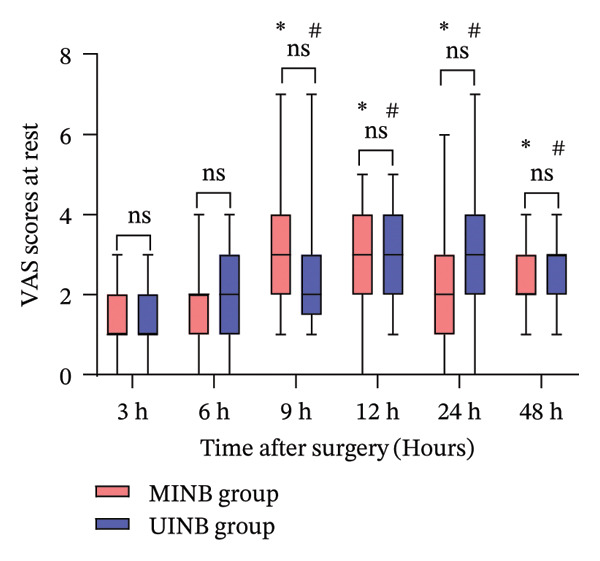
(a)

**Figure:**
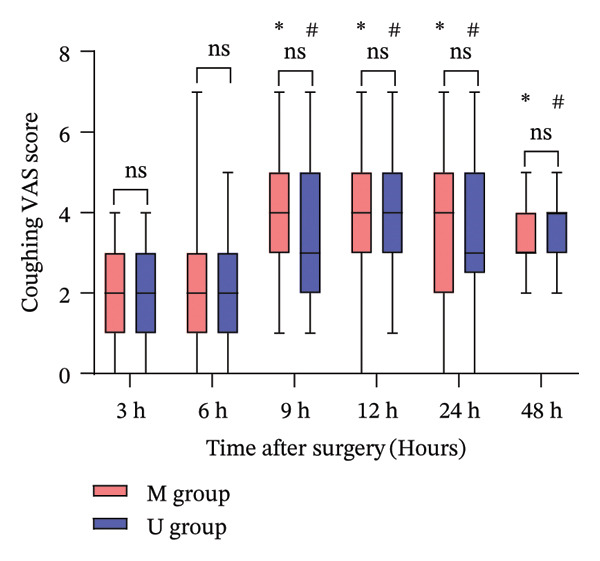
(b)

**TABLE 2:** Secondary outcomes.

Outcome	MINB group (*n* = 35)	UINB group (*n* = 37)	*p* value
Puncture success rate (*n*, %)	35 (100)	37 (100)	> 0.999
Puncture time (mins)	4.6 ± 1.3	13.2 ± 1.6	< 0.001^∗^
Ropivacaine dosage (mg)	50 ± 0.0	61.9 ± 4.6	< 0.001^∗^
Pleural injury (*n*, %)	0 (0)	3 (8.1)	0.240
Vascular injury (n, %)	0 (0)	6 (16.2)	0.025^∗^
48‐h sufentanil dosage (μg)	62.9 ± 13.6	67.3 ± 10.1	0.132
48‐h total presses of PCIA (times)	9.9 ± 3.7	11.2 ± 2.4	0.101
First analgesic requirement (hours)	5.9 ± 1.2	5.5 ± 1.4	0.152
Rescue analgesia (0 time/1 time/2 times)	28/6/1	32/4/1	0.751
PONV scores (0/1/2/3)	29/5/1/0	35/1/1/0	0.150
Patient satisfaction score	7 (7–9)	8 (7.5–8)	0.073

*Note:* Data are expressed as mean ± SD, median (IQR), or number of patients (%).

Abbreviations: MINB, ultrasound‐guided modified intercostal nerve block; UINB, ultrasound‐guided intercostal nerve block; PCIA, patient‐controlled intravenous analgesia; PONV, postoperative nausea and vomiting.

^∗^Statistical significance (*p* < 0.05).

## 4. Discussion

The present study demonstrates that single‐injection MINB is noninferior to multipoint UINB for postoperative analgesia in pediatric patients undergoing MIRPE with a single bar. MINB demonstrates substantial clinical benefits, including a 65% reduction in block performance time (4.6 vs. 13.2 min) and a 19% decrease in ropivacaine dosage (50.0 vs. 61.9 mg). It also shortens the onset time of anesthesia and eliminates vascular injuries (0% vs. 16.2%). Furthermore, MINB achieves efficacy comparable to UINB across multiple outcomes: 48‐h pain scores, opioid consumption, need for rescue analgesia, incidence of analgesia‐related adverse events, and patient satisfaction.

Post‐MIRPE pain arises from a multifactorial pathogenesis. Contributing elements can be attributed to the mechanical pressure of the intrathoracic bar, direct incisional trauma, persistent muscular hypertonicity, and the activation of local and systemic inflammatory pathways following the procedure [1, 3, 4, 9, 26]. The primary pain signals in patients arise from the intercostal nerves and their branches, with somatic nociceptive afferents playing the central role in transmitting this sensation [16, 17, 27]. Currently, no cadaveric anatomical studies have been conducted to elucidate the analgesic mechanism and local anesthetic diffusion pattern of MINB [21]. MINB targets the injection site at the intersection of the fascial plane formed by the deep surface of the serratus anterior muscle and the superficial layer of the external intercostal muscles with the inferior margin of the rib. This approach is postulated to establish an optimal depot for local anesthetic diffusion that may simultaneously access the main intercostal nerve trunk and its lateral cutaneous branches [21, 28–30]. This dual neural blockade mechanism—integrating serratus anterior plane block with intercostal nerve block—provides the physiological basis for the noninferiority of MINB to UINB in managing post‐MIRPE pain.

Technical differences during procedural implementation account for the advantages of MINB over UINB. UINB can be administered along the entire length of any rib, with the specific site selected according to surgical approach; consequently, patient positioning may vary (prone, lateral decubitus, or supine) [16, 17]. This study adopted Luo et al. [10] UINB protocol, performing injections near the midaxillary line under supine positioning to ensure blockade of the lateral cutaneous branches of the intercostal nerves. Anatomical studies indicate that intercostal nerves typically give rise to lateral cutaneous branches near the midaxillary line, which perforate the intercostal muscles and serratus anterior muscle to innervate the lateral thoracic wall [4, 15, 17]. Given that post‐MIRPE pain often involves multiple dermatomes (due to traction from sternal bar placement during pectus excavatum correction [4]), blocks were administered at three intercostal spaces adjacent to bilateral incisions [10]. In pediatric patients with extensive deformities requiring multiple sternal bars, blockade at five to eight intercostal levels was occasionally necessary [2, 31].

In contrast, MINB requires only a single injection adjacent to each incision. Based on cadaveric studies of fascial plane blocks and our clinical experience, accurate needle‐tip placement with the bevel oriented downward enables diffusion of minimal local anesthetic volume to cover both the deep intercostal nerve trunks and multiple intercostal regions along the deep fascial plane of the serratus anterior muscle (in pediatric patients, the looser musculofascial architecture may facilitate a wider spread for a given volume) [19, 20, 28–30]. Given the technical considerations, the observed 65% reduction in block time with MINB aligns with expectations, which also leads to a shorter total anesthesia duration. Reducing anesthesia time serves a dual purpose: It directly decreases neurodevelopmental risks in pediatric patients while also improving workflow efficiency to accommodate busy operating room schedules [32, 33].

Current evidence regarding the diffusion mechanisms and optimal local anesthetic volume for MINB remains limited. The local anesthetic volume for MINB (10 mL) was determined based on recommended doses for pediatric fascial plane blocks (0.3–0.5 mL/kg) [34], considering a mean prestudy pilot patient weight of 35 kg. Conversely, the maximum volume administered in the UINB group reached 30 mL (75 mg ropivacaine). Given the rich vascularization of the intercostal space and rapid systemic absorption of local anesthetics in children, this volume raises concerns regarding potential local anesthetic systemic toxicity (LAST) [35].

Additionally, since UINB targets sites adjacent to the pleura and vasculature, reported rates of asymptomatic pneumothorax reach 1.4%–3.4% per attempt under landmark guidance, though ultrasound reduces this risk [17, 36]. In this study, intraoperative thoracoscopy revealed a pleural hematoma in 16.2% (6/37) and direct pleural puncture by the needle in 8.1% (3/37) of patients in the UINB group. By contrast, the superficial target plane of MINB resulted in no such complications.

The differential analgesic efficacy of MINB between adult and pediatric populations reveals clinically significant divergence. Although MINB provided substantial opioid‐sparing and pain reduction in adult MITS patients versus controls, a less dramatic effect was observed in pediatric cases following MIRPE [19, 20]. The observed discrepancy can be attributed to methodological design and pathophysiological differences. A key distinction lies in the control groups: The adult trial employed a placebo‐controlled design, which clearly isolated MINB’s absolute analgesic benefits. In contrast, the pediatric study used an active comparator (UINB), a design that directly influences how outcomes are interpreted. However, contemporary pediatric MIRPE guidelines mandate multimodal analgesia incorporating regional techniques, necessitating a noninferiority design comparing MINB with UINB [1]. Second, although no studies directly compare postoperative pain intensity and distribution between adult MITS and pediatric MIRPE, existing evidence indicates that MITS reduces pain and enhances recovery versus open thoracotomy in adults [37]. Conversely, MIRPE elicits higher postoperative pain intensity than open repair in children [5, 6]. Nagasao et al. [26] demonstrated that pediatric MIRPE pain distributes diffusely across the anterolateral chest wall. We therefore hypothesize that the attenuated analgesic efficacy of MINB in pediatric MIRPE, compared to adult studies, may arise from higher baseline pain intensity inherent to MIRPE surgery.

Despite these advances, VAS monitoring revealed a significant increase in pain scores from 9 h onward relative to the 3‐h baseline in both groups (Figure 4). The observed data revealed a rising trend in pain, which aligns with the established pattern of increasing pain levels over the initial 120‐h period following pediatric MIRPE [11]. Furthermore, the waning analgesic effect of ropivacaine likely contributes to the reduced pain control over time [38]. The observed analgesic timeline is consistent with published evidence. Luo et al. [10] demonstrated that single‐injection UINB significantly improved pain scores within 6 h post‐MIRPE versus PCIA, with Lukosiene et al. [31] confirming its parallel capacity to significantly curb opioid consumption in this early window. Given this dual mechanism, we believe extending MINB’s therapeutic window is a crucial next step. Our team will therefore focus on three key approaches: first, adding adjuvant dexamethasone to leverage its perineural anti‐inflammatory effect for prolonged blockade [39]; second, adopting sustained‐release formulations like liposomal bupivacaine, which has shown promise in providing up to 72 h of analgesia in children [40]; and third, implementing continuous local anesthetic infusion via catheter.

Our findings should be considered in the context of several limitations. The notable difference in puncture sites between techniques may have compromised blinding, as clinicians could potentially discern the group allocation, introducing a risk of measurement bias. Additionally, the age requirement for VAS scoring restricted our enrollment to children 8 years and older [23], leaving the efficacy of MINB in younger patients unexplored. The study also exclusively focused on patients receiving a single bar during MIRPE, and its applicability to procedures involving multiple bars remains unknown. Finally, the single‐center design and limited sample size necessitate confirmation through future, larger multicenter investigations.

## 5. Conclusion

For children undergoing single‐bar MIRPE, MINB provides noninferior postoperative analgesia relative to UINB while offering substantial advantages: a 65% reduction in block placement time, a 19% decrease in ropivacaine dosage, shortened anesthesia duration, and enhanced safety (absence of vascular injuries). These findings position MINB as a safer and technically streamlined alternative to UINB for managing pain after this procedure.

## Funding

No funding was received for this study.

## Ethics Statement

This study was approved by the Medical Ethics Committee of Anhui Provincial Children’s Hospital (Approval No: EYLL‐2022–039).

## Consent

Written informed consent was obtained from the legal guardians of all pediatric participants (with age‐appropriate participants also informed) prior to enrollment, following full disclosure of the trial protocol, risks, benefits, and withdrawal rights (without medical care impact). All procedures complied with the approved protocol.

## Conflicts of Interest

The authors declare no conflicts of interest.

## Data Availability

The data that support the findings of this study are available from the corresponding author upon reasonable request.
